# Altered CYP19A1 and CYP3A4 Activities Due to Mutations A115V, T142A, Q153R and P284L in the Human P450 Oxidoreductase

**DOI:** 10.3389/fphar.2017.00580

**Published:** 2017-08-25

**Authors:** Sameer S. Udhane, Shaheena Parween, Norio Kagawa, Amit V. Pandey

**Affiliations:** ^1^Department of Pediatric Endocrinology, Diabetology and Metabolism University Children’s Hospital Bern, Bern, Switzerland; ^2^Department of Clinical Research, University of Bern Bern, Switzerland; ^3^School of Medicine, Nagoya University Nagoya, Japan

**Keywords:** POR, P450 oxidoreductase, CYP19A1, CYP3A4, polymorphisms, protein–protein interaction, cytochrome P450

## Abstract

All cytochromes P450s in the endoplasmic reticulum rely on P450 oxidoreductase (POR) for their catalytic activities. Mutations in POR cause metabolic disorders of steroid hormone biosynthesis and affect certain drug metabolizing P450 activities. We studied mutations A115V, T142A, Q153R identified in the flavin mononucleotide (FMN) binding domain of POR that interacts with partner proteins and P284L located in the hinge region that is required for flexibility and domain movements in POR. Human wild-type (WT) and mutant POR as well as CYP3A4 and CYP19A1 proteins in recombinant form were expressed in bacteria, and purified proteins were reconstituted in liposomes for enzyme kinetic assays. Quality of POR protein was checked by cytochrome c reduction assay as well as flavin content measurements. We found that proteins carrying mutations A115V, T142A located close to the FMN binding site had reduced flavin content compared to WT POR and lost almost all activity to metabolize androstenedione via CYP19A1 and showed reduced CYP3A4 activity. The variant P284L identified from apparently normal subjects also had severe loss of both CYP19A1 and CYP3A4 activities, indicating this to be a potentially disease causing mutation. The mutation Q153R initially identified in a patient with disordered steroidogenesis showed remarkably increased activities of both CYP19A1 and CYP3A4 without any significant change in flavin content, indicating improved protein–protein interactions between POR Q153R and some P450 proteins. These results indicate that effects of mutations on activities of individual cytochromes P450 can be variable and a detailed analysis of each variant with different partner proteins is necessary to accurately determine the genotype-phenotype correlations of POR variants.

## Introduction

Cytochrome P450 proteins metabolize a wide range of drugs, steroids and xenobiotics (Schuster and Bernhardt, 2007; Omura, 2010; Zanger and Schwab, 2013). There are two distinct types of cytochrome P450 proteins in humans (Omura, 2010). Type 1 P450s, that are located in the mitochondria, are primarily involved in the metabolism of steroids and use adrenodoxin/adrenodoxin reductase proteins as redox partners (Omura, 2006; Zalewski et al., 2016). The type 2 P450s, located in the endoplasmic reticulum, rely on a single redox partner, the cytochrome P450 oxidoreductase (POR, NCBI# NP_000932, UniProt# P16435), for supply of electrons (Lu et al., 1969). POR is a flavoprotein that contains both the flavin mononucleotide (FMN) and the flavin adenine dinucleotide (FAD) and supplies electrons to cytochromes P450 and other proteins (Matsubara et al., 1976; Nagao et al., 1983; Guengerich, 2005). The structure of the FMN binding domain of human POR has been determined by x-ray crystallography (Zhao et al., 1999) and a more recent open conformation structure of POR shows potential interaction possibilities for redox partners (Aigrain et al., 2009).

**FIGURE 1:**
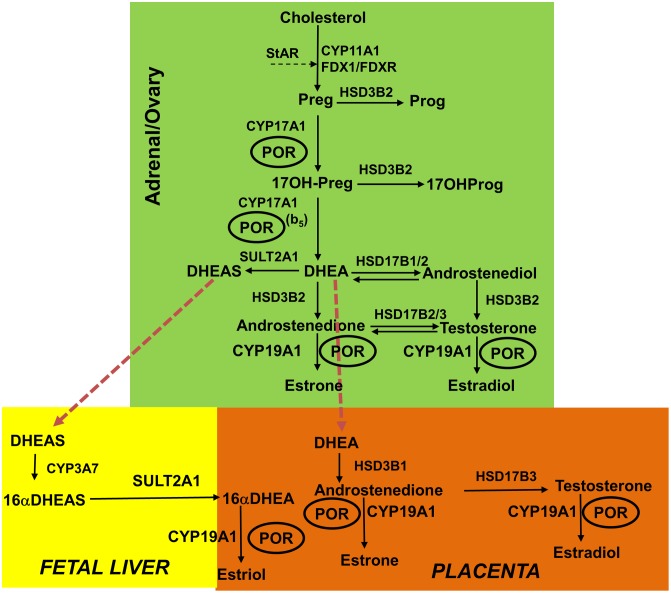
Role of POR in the steroid hormone biosynthesis. In the first steps of steroid biosynthesis, cholesterol is transported to mitochondrion by steroidogenic acute regulatory protein (StAR). Then, inside the mitochondria, the cholesterol is metabolized by CYP11A1 to pregnenolone, and adrenodoxin and adrenodoxin reductase act as the redox mediators in this reaction. The pregnenolone produced in the mitochondria is further metabolized in the endoplasmic reticulum to downstream products 17OHPreg, DHEA, androstenedione or androstenediol; and then this process continues toward the production of testosterone in the Leydig cells of the testes. Testosterone is further metabolized to dihydrotestosterone (DHT) in the genital skin. In the placenta, DHEA is converted to androstenedione and then to testosterone, which is further metabolized to estrogens. In fetal liver, inactive precursor DHEAS is converted to 16αDHEAS, which gets converted to 16αDHEA in placenta to produce estrogens. The estrogen biosynthesis process requires CYP19A1 activity that is dependent on POR. The deficiency in POR can alter the enzymatic reactions of many cytochrome P450 enzymes and these changes can be variable based on structural properties of the mutation and nature of the redox partner. CYP11A1, P450scc, cholesterol side-chain cleavage enzyme; StAR, steroidogenic acute regulatory protein; FDX1, Adrenodoxin; FDXR, NADPH adrenodoxin oxidoreductase; CYP17A1, P450c17, 17α-hydroxylase/17,20-lyase; HSD3B2, 3βHSD2, 3β-hydroxysteroid dehydrogenase, type 2; CYB5, cytochrome b_5_; POR, P450 oxidoreductase; HSD17B3, 17βHSD3, 17β-hydroxysteroid dehydrogenase, type 3. Full steroid names: 17OHPreg, 17-hydroxypregnenolone; 17OHProg, 17-hydroxyprogesterone; 17OH-DHP, 17-hydroxydihydroprogesterone (5α-pregnan-3α,17α-ol-20-one); DHEA, dehydroepiandrosterone. Copyright copyright 2017 Udhane, Parween, Kagawa and Pandey.

P450 oxidoreductase deficiency (PORD, OMIM: 613537 and 201750) is a form of congenital adrenal hyperplasia, initially described in patients with altered steroidogenesis (Flück et al., 2004) followed by several reports with a broad spectrum of disorders (Adachi et al., 2004; Arlt et al., 2004; Pandey and Flück, 2013; Flück and Pandey, 2014; Pandey and Sproll, 2014; Burkhard et al., 2017). Sequencing of the POR gene revealed mutations in patients with disorders of steroid biosynthesis (Flück et al., 2004; Miller et al., 2004; Pandey et al., 2004). Afterward, many other laboratories reported mutations in POR in patients with steroidogenic disorders and/or bone malformation syndromes (Adachi et al., 2004; Arlt et al., 2004; Fukami et al., 2005; Pandey and Flück, 2013; Riddick et al., 2013). While initial studies on POR mutations focused on steroid metabolism, effects on other partner proteins like heme oxygenase and drug metabolizing P450s have also been studied (Agrawal et al., 2008, 2010; Flück et al., 2010; Nicolo et al., 2010; Pandey et al., 2010). Large scale sequencing projects have identified many variations in the POR gene, in several different human sub-populations, and multiple variants have been implicated in altered drug metabolism (Gomes et al., 2008, 2009; Huang et al., 2008; Agrawal et al., 2010; Nicolo et al., 2010; Pandey et al., 2010). Since POR is involved in multiple steroid biosynthesis reactions catalyzed by cytochrome P450 proteins, a complex disorder of steroidogenesis is expected from mutations in POR (**Figure 1**). Many POR variants found in patients as well as normal population have been tested for enzymatic activities (Adachi et al., 2004; Arlt et al., 2004; Fukami et al., 2005, 2006; Homma et al., 2006; Pandey, 2006; Huang et al., 2008; Sim et al., 2009). While mutations in POR can be found in all regions of the protein, based on analysis of previously identified mutations, some general observations can be made. Mutations found in the co-factor binding sites (FMN, FAD and NADPH) generally result in a severe form of the disease with the mutations causing loss of FMN or FAD showing most severe effects on activities of all supported redox partners. We have previously shown that aromatase (CYP19A1) activity responsible for conversion of androgens to estrogens (**Figure 1**) is more susceptible to changes in NADPH binding site mutations in POR (Pandey et al., 2007; Flück et al., 2011; Flück and Pandey, 2017). The studies described in this report were conducted by embedding POR and P450 proteins in liposomes followed by removal of detergents. In the current study, we have investigated effects of some mutations in the FMN binding domain and the hinge region of POR that facilitate interactions with redox partner proteins, including P450s, to transfer electrons from NADPH.

## Materials and Methods

### Recombinant Expression of POR and Membrane Purification

The WT and variant POR (NCBI# NP_000932, Uniprot# P16435) proteins were expressed in bacteria using the expression constructs described previously (Huang et al., 2005, 2008; Pandey et al., 2007; Nicolo et al., 2010). The protocol for expression of N-27 form of POR variants and subsequent membrane purification is described and adopted from our previous publications (Huang et al., 2005; Pandey et al., 2007, 2010; Parween et al., 2016; Flück and Pandey, 2017). The cDNAs for WT or mutant POR in pET22b were transformed into *Escherichia coli* BL21(DE3), single colonies were selected for growth on ampicillin and grown in terrific broth pH 7.4 supplemented with 40 mM FeCl_3_, 4 mM ZnCl_2_, 2 mM CoCl_2_, 2 mM Na_2_MoO_4_, 2 mM CaCl_2_, 2 mM CuCl_2_, 2 mM H_3_BO_3_, 0.5 mg/ml riboflavin, 100 μg/ml carbenicillin at 37°C to an optical density (OD) 600 nm of 0.6 and temperature was reduced to 25°C for 16 h. The bacterial cells were collected by centrifugation, washed with PBS and suspended in 100 mM Tris–acetate (pH 7.6), 0.5 M sucrose, and 1 mM EDTA and treated with lysozyme (0.5 mg/ml) and EDTA (0.1 mM [pH 8.0]) at 4°C for 1 h with slow stirring to generate spheroplasts. The spheroplasts were pelleted by centrifugation at 5000 × *g* for 15 min; and suspended in 100 mM potassium phosphate (pH 7.6), 6 mM MgOAc, 0.1 mM DTT, 20% (v/v) glycerol, 0.2 mM PMSF, and 0.1 mM DNase I; and disrupted by sonication. A clear lysate devoid of cellular debris was obtained by centrifugation at 12,000 × *g* for 10 min, and then the membranes were collected by centrifugation at 100,000 × *g* for 60 min at 4°C. Membranes were suspended in 50 mM Potassium phosphate buffer (pH 7.8) and 20% (v/v) glycerol and stored at -70°C. Protein concentration was measured by RC-DC protein assay (Protein Assay Dye Reagent, Bio-Rad, Hercules, CA, United States) and POR content in membrane proteins was quantitated by western blot analysis.

### Quantification of POR Content in the Bacterial Membranes by Western Blot Analysis

For Western blots, 1 μg of WT and POR bacterial membrane proteins were separated on SDS–PAGE gel and blotted on to polyvinyldifluoride (PVDF) membranes. Blots were probed with a rabbit polyclonal antibody against wild-type human POR from Genscript (Genscript, Piscataway, NJ, United States) at a dilution of 1:1000. We used a secondary goat anti-rabbit antibody that was labeled with a phthalocyanine infrared dye (IRDye 700DX, LI-COR Bioscience Inc., Lincoln, NE, United States) at a dilution of 1:10000. Signals were detected with the green fluorescent channel (700 nm) on an Odyssey Infrared Imaging System (LI-COR Bioscience Inc., Lincoln, NE, United States), and bands were quantitated using Odyssey software. POR content of each membrane preparation was measured, and all samples were normalized against purified wild-type POR used as standard. In all experiments the normalized amount of POR content was used in each experiment for all mutants as well as WT protein.

### Cytochrome c Reduction Assay by WT and Mutant POR

The cytochrome c reduction by bacterially expressed WT or mutant POR was assayed as described previously by measuring the change in absorbance at 550 nm (ε = 21.1 cm^-1^ mM^-1^) (Guengerich et al., 2009). Briefly, the reaction was performed in 96-well plates, in triplicate, with 5 μg of POR membrane preparation in each well, using a microplate reader (Spectramax M2e, Molecular Devices, Sunnyvale, CA, United States). The NADPH concentration was fixed at 100 μM and different concentrations of cytochrome c (0.6–80 μM) were used. The change in absorbance at 550 nm was monitored against time. Data were fitted based on Michaelis–Menten kinetics (Michaelis and Menten, 1913) using GraphPad Prism (GraphPad Software, La Jolla, CA, United States) to determine the Vmax and Km.

### Flavin Content Analysis of WT and Mutant POR

To differentiate the conformational changes and effects of co-factor binding, we evaluated the relative flavin content in POR variants as the activity of POR is affected by the binding of cofactors FMN and FAD. Protein bound flavin molecules were released by thermal denaturation of POR proteins (Faeder and Siegel, 1973). Flavin content of WT and mutant POR proteins (100 μg/ml) was determined by heating protein samples at 95°C for 10 min in the dark, followed by centrifugation at 14000 × *g* for 10 min to remove the coagulated protein. The FMN and FAD ratio was determined by measurement of fluorescence of the supernatant at pH 7.7 and pH 2.6 (excitation at 450 nm, emission at 535 nm) (Faeder and Siegel, 1973) using commercially available FMN and FAD as standards (Sigma–Aldrich, Basel, Switzerland).

### CYP19A1 Expression and Purification

The CYP19A1 vector for bacterial expression was transformed in *E. coli* BL21(DE3) cells and the recombinant protein was expressed and purified following previously published protocols (Kagawa et al., 2003; Kagawa, 2011), with slight modifications. Briefly, a single transformed colony was selected for protein expression at 25°C. After 4 h of incubation, 1 mM δ-aminolevulinic acid (a heme precursor) and 4 mg/ml arabinose (for induction of molecular chaperones GroEL/GroES) were added to the culture and further incubated for 20 h. Cells were harvested and spheroplasts were prepared with 0.2 mg/ml lysozyme in 50 mM Tris–Acetate (pH 7.6), 250 mM sucrose and 0.5 mM EDTA and stored at -80°C. For protein purification, spheroplasts were lysed using 10XCellLytic B (Sigma–Aldrich) in buffer containing 100 mM potassium phosphate (pH 7.4), 500 mM sodium acetate, 0.1 mM EDTA, 0.1 mM DTT, 20% glycerol, and 1 mM PMSF. The cell lysate was centrifuged and the supernatants were pooled for purification by Ni^2+^ affinity chromatography. Purification was performed at 4°C and protein concentration after the dialysis was determined by DC protein assay (Protein Assay Dye Reagent, Bio-Rad, Hercules, CA, United States) using BSA as standard.

### Aromatase Activity Measurement in Reconstituted Liposome System

**FIGURE 2:**
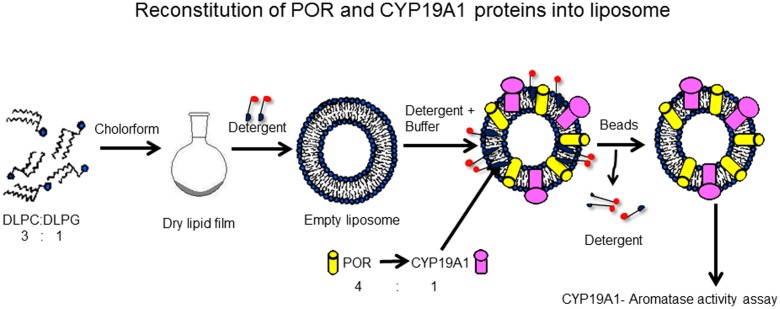
Reconstitution of POR and CYP19A1 proteins into liposomes. Lipids (DLPC: DLPG) were dissolved in chloroform and kept overnight for drying in a fume hood. Next day, dried lipids were solubilized in detergent to prepare liposomes. The CYP19A1 and POR membranes were added to the liposomes and the protein-liposome mixture was incubated for 1 h with shaking to incorporate the proteins into the liposomes. After 1 h, biobeads were added to remove the detergent, and samples were incubated for another 1 h. After incubation, the mixture was cleared by centrifugation and the supernatant was used for assay of CYP19A1 activity. Copyright copyright 2017 Udhane, Parween, Kagawa and Pandey.

Purified recombinant CYP19A1 using the bacterial expression system was used to test the effect of POR variants to support the aromatase activity of CYP19A1. Standard tritiated water release assay for the CYP19A1 activity was performed in a reconstituted liposome system using androstenedione as substrate. Bacterial membranes containing POR and purified CYP19A1 were reconstituted into DLPC-DLPG liposomes. The liposomes were prepared as described in **Figure 2**. Aromatase activity was measured by the tritiated water release assay originally described by Lephart and Simpson (1991) with modifications as described by us previously (Pandey et al., 2007; Flück and Pandey, 2017) using a reconstituted lipid-CYP19A1-POR system and androstenedione as the substrate. Reaction mixture consisted of 100 pmol of CYP19A1, 400 pmol of POR, 100 mM NaCl and ^3^H labeled androstenedione ([1β-^3^H(N)]-andros-tene-3,17-dione; ∼20,000 cpm) in 100 mM potassium-phosphate buffer (pH 7.4). Different concentrations (10–1000 nM) of androstenedione were used for kinetic analysis. The catalytic reaction was initiated by the addition of 1 mM NADPH and the reaction tube was incubated for 1 h under shaking. Data were fitted based on Michaelis–Menten kinetics using GraphPad Prism (GraphPad Software, La Jolla, CA, United States).

### CYP3A4 Activity Measurement in Reconstituted Liposome System

To compare the activities of CYP19A1 with another steroid binding cytochrome P450, we tested the effect of POR mutations to support the enzyme activity of CYP3A4. The activity of the major drug metabolizing enzyme CYP3A4 supported by WT or mutant POR was tested using the fluorogenic substrates [BOMCC (7-Benzyloxy-4-trifluoromethylcoumarin) and DBOMF dibenzylmethylfluorescein] (Invitrogen Corp, Carlsbad, CA, United States) as described earlier (Flück et al., 2010). The purified CYP3A4 (CYPEX, Dundee, Scotland, United Kingdom) was used to test the activities of the POR variants using 20 μM BOMCC or 5 μM DBOMF as substrate (the apparent Km value of CYP3A4 for BOMCC, 10 μM, DBOMF 2.5 μM were derived from pilot experiments using WT POR and CYP3A4). *In vitro* CYP3A4 assays were performed using a reconstituted liposome system consisting of WT/mutant POR, CYP3A4 and cytochrome b_5_ at a ratio of 4:1:1 (POR:CYP3A4:b_5_). Reconstitution of liposomes was carried out similarly as described before. The final assay mixture consisted of liposomes and proteins (80 pmol POR: 20 pmol CYP3A4: 20 pmol b_5_), 2.5 mM MgCl_2_, 2.5 μM GSH and 20 μM BOMCC or 5 μM DBOMF in 50 mM HEPES buffer and the reaction volume was 200 μl. The catalytic reaction was initiated by addition of NADPH to 1 mM final concentration and fluorescence was monitored on a Spectramax M2e plate reader (Molecular Devices, Sunnyvale, CA, United States) at an excitation wavelength of 415 nm and emission wavelength of 460 nm for BOMCC and at an excitation wavelength of 490 nm and emission wavelength of 520 nm for DBOMF.

### Statistical Analysis

Data are presented as mean standard errors of mean (SEM) in each group or replicates. Differences within the subsets of experiments were analyzed using Student’s *t*-test with GraphPad Prism (GraphPad Software Inc., CA, United States). *P*-values less than 0.05 were considered statistically significant.

### 3D Protein Models

Three dimensional structural models of POR (NCBI# NP_000932) proteins were obtained from protein structure database^1^. We used the structures of the FMN binding domain of human POR (PDB # 1B1C) as well as an open structure of POR protein (PDB# 3FJO) to analyze the location of amino acids described in this report (Zhao et al., 1999; Aigrain et al., 2009). Structure models were drawn using the software Pymol^2^ and rendering of images was performed with POVRAY^3^.

## Results

### Cytochrome c Reduction Assay

**FIGURE 3:**
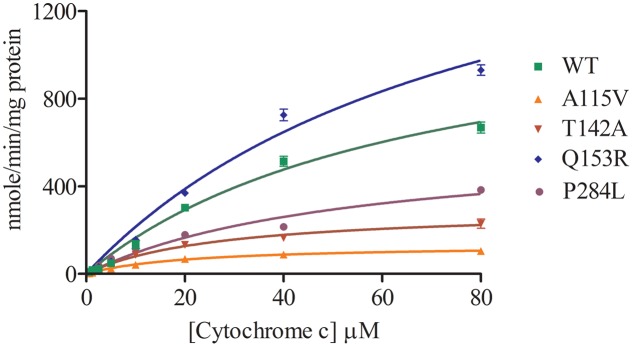
Cytochrome c reduction assay with WT and mutant POR. Cytochrome c reduction assays was performed with the WT and variant forms of POR. Kinetic assay relied on the changes in absorbance at 550 nm when oxidized cytochrome c is converted into reduced cytochrome c. Data were fitted as Michaelis–Menten kinetics model using GraphPad Prism. The calculated Km and Vmax values are summarized in **Table 1**.

Cytochrome c reduction assay with WT and POR variants was performed to assess the basic quality and general catalytic efficiency of POR as well as internal electron transfer in POR. As compared to WT POR, 40–70% loss of activity in reducing cytochrome c was observed with POR variants A115V, T142A and P284L (**Figure 3** and **Table 1**). This loss of activity indicates that these mutations affect electron transport in POR through flavins to cytochrome c since reduction of cytochrome c requires the participation of both FAD and FMN as do the cytochromes P450. Interestingly, Q153R showed 25% higher cytochrome c reduction activity than WT POR (**Table 1**).

**Table 1:** Kinetic parameters for activities of cytochrome c reduction and CYP19A1 activity supported by WT and mutant POR.

	Km, cytochrome c (μM)	Vmax nmol/min/mg	Vmax/Km	% WT
**Cytochrome c reduction assay**				
	67.4 ± 11.2	1279 ± 123.7	19	100
A115V	20.9 ± 11.1*	133.4 ± 2.8*	6.4	34
T142A	27.0 ± 13.4*	298.1 ± 15.4*	11	58
Q153R	81.5 ± 18.2*	1968 ± 270.2*	24.2	127
P284L	53.7 ± 10.5*	610 ± 63.5*	11.4	60

	**Km androstenedione (nM)**	**Vmax pmol/min/nmol**	**Vmax/Km**	**% WT**

**CYP19A1; aromatase (androstenedione to estrone)**				
WT	80 ± 14.5	0.72 ± 0.03	0.0090	100
A115V	nd	nd	nd	–
T142A	308.4 ± 121.5*	0.11 ± 0.01*	0.0004	5
Q153R	65.62 ± 11.38*	0.86 ± 0.03*	0.0132	147
P284L	82.4 ± 62.5*	0.039 ± 0.008*	0.0005	5

*For the cytochrome c reduction assay, the NADPH concentration was fixed at 100 μM and different concentrations of cytochrome c (0.6–80 μM) were used. For the conversion of androstenedione to estrone, variable concentrations (10–1000 nM) of androstenedione were used for kinetic analysis in presence of 1 mM NADPH. Data are presented as mean ± SEM of independent replicates. ^∗^Indicates *p*-values < 0.05. nd: not detectable*.

### Flavin Content

**FIGURE 4:**
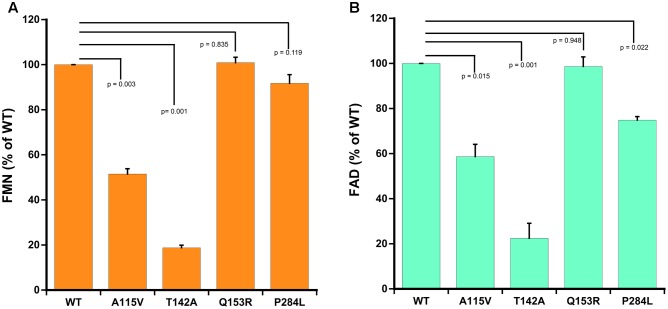
Flavin content of WT and mutant POR proteins. Flavin content was measured by boiling protein samples under different buffer conditions. Relative fluorescence unit (RFU) of the flavins released from POR variants obtained at pH 7.7 (F_7.7_) and pH 2.6 (F_2.6_) were plotted. RFU of WT was set as hundred percent. **(A)** Shows FMN measurements and **(B)** shows FAD measurements. *p*-values for significance analysis compared to wild-type sample are indicated above the bars. Data are presented as mean ± SEM of three replicates.

As compared to WT POR, a 40–50% decrease in flavin content of A115V mutant was observed (0.52 mol FMN/mol of POR and 0.59 mol FAD/mol of POR). Both the FMN as well as the FAD binding was severely affected due to T142A mutation with approximately 80% loss of relative flavin content as compared to WT (0.19 mol FMN/mol of POR and 0.22 mol FAD/mol of POR) (**Figure 4**). The total flavin content of the gain of function mutation Q153R (1 mol FMN/mol of POR and 0.99 mol FAD/mol of POR) was comparable to WT and for the loss of function mutation P284L the FMN content was similar to WT (0.91 mol FMN/mol of POR) while the FAD content was reduced by 25% (0.75 mol FAD/mol of POR) suggesting that these mutations do not severely affect either the FMN or the FAD binding to POR (**Figure 4**).

### CYP19A1-Aromatase Activity

**FIGURE 5:**
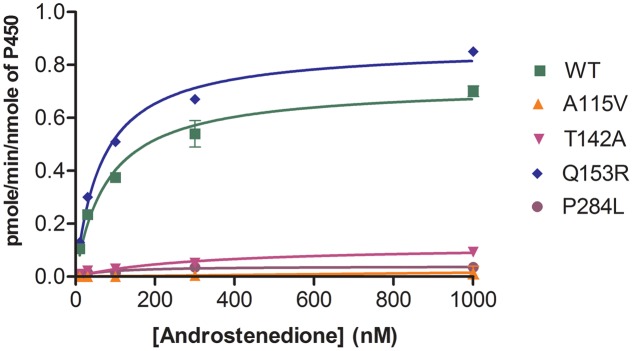
Aromatase activity supported by WT and mutant POR proteins. Bacterially expressed purified recombinant CYP19A1 and POR membranes were incorporated into liposomes and their activity to convert [^3^H] labeled androstenedione to estrone was tested by the tritiated water release assay. Data were fitted with Michaelis–Menten kinetics model using GraphPad Prism. The calculated Km and Vmax values are summarized in **Table 1**.

The POR variants A115V, T142A and P284L showed almost complete loss of CYP19A1 activity (**Figure 5** and **Table 1**). For the T142A variant, the apparent Km for androstenedione was increased fourfold as compared to WT POR suggesting that T142A mutation affects either substrate interaction of CYP19A1 or the CYP19A1-POR interaction. The apparent Km with P284L variant was comparable to that of WT POR but the apparent Vmax was reduced by 85%. Interestingly, POR variant Q153R showed 47% higher value of Vmax/Km compared to WT POR.

### CYP3A4 Enzyme Activity

**FIGURE 6:**
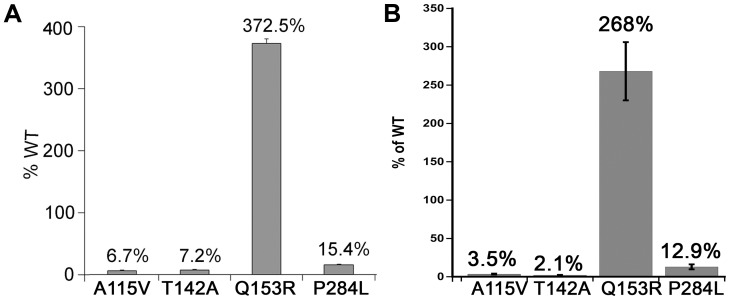
CYP3A4 activity supported by WT and mutant POR proteins. CYP3A4 enzyme activity assays were performed to compare WT and mutant POR using either **(A)** 20 μM BOMCC or **(B)** 5 μM DBOMF as substrate. Activity with the WT POR was set as hundred percent and results are shown as percentage of WT activity. Data are presented as mean ± SEM of three independent replicates.

The A115 V and T142A variants of POR showed 2–10% activity compared to WT (**Figure 6**). In human POR residues A115V and T142A are directly involved in FMN binding. The POR variant P284L resulted in 12–15% of CYP3A4 activity (**Figure 6**). The CYP3A4 activity of POR variant Q153R was nearly three times more than wild-type (WT) POR (**Figure 6**).

## Discussion

P450 oxidoreductase is a membrane associated protein that binds NADPH, causing a conformation change that brings NADPH and FAD close together to transfer electrons. Afterward, further conformation changes cause a closing of the POR structure and brings FAD and FMN closer for electron transfer from FAD to FMN. The FMN binding domain of POR interacts with P450s and other partner proteins through charge pair interactions involving arginine and lysine residues on P450s and aspartate and glutamate residues on POR. Considering the importance of FMN binding domain of POR in protein–protein interactions we investigated the effects of mutations in this region of POR for enzymatic activities on partner proteins. The mutations studied in this report are either located close to the FMN binding site of POR (A115V, T142A and Q153R) or in the putative hinge region (P284L) required for flexibility of POR to interact with redox partners. Using WT and variant POR and P450 proteins expressed in bacteria, we determined the enzymatic activities for metabolism of androstenedione by CYP19A1 and compared them with CYP3A4 activities which can bind and metabolize testosterone. Some very interesting and surprising findings were made from this study, including the severe effect of POR variant A115V which was previously suspected as a polymorphism (rs 199634961, *POR^∗^11*) and shows variable activities (Huang et al., 2005; Agrawal et al., 2008, 2010; Pandey et al., 2010; Pandey and Flück, 2013; Burkhard et al., 2017). The POR variants P284L was found to result in severe loss of activities while mutation Q153R showed an activating effect. These results provide further insights into the interactions of POR with its redox partner proteins and help in establishing the genotype-phenotype correlations of POR variants and their metabolic effects. Loss of flavins could explain differences in activities for some mutations. Since the A115 and T142 residues are near the FMN binding site, a loss of FMN binding could be expected. However, both these mutants also showed loss of FAD binding indicating that binding of FMN may influence FAD binding. A similar relationship of FAD binding to FMN binding has been observed by Shen et al. (1989) in experiments with rat POR. Several different proposals could be made to explain this observation. It is likely that binding of FMN is required for recognition of FAD by POR. An FMN dependent change if affinity of POR for FAD is possible (Shen et al., 1989). The observation of Kurzban and Strobel (1986) that FAD comes of more readily from POR compared to FMN seems to support the hypothesis that affinity of POR for FMN is higher. Mutations in the FAD binding domain of POR result in complete loss of activities (Flück et al., 2004; Pandey et al., 2004; Huang et al., 2005; Pandey and Flück, 2013). For some POR mutations a rescue of activities may be possible by flavin supplementation (Marohnic et al., 2010; Nicolo et al., 2010).

**FIGURE 7:**
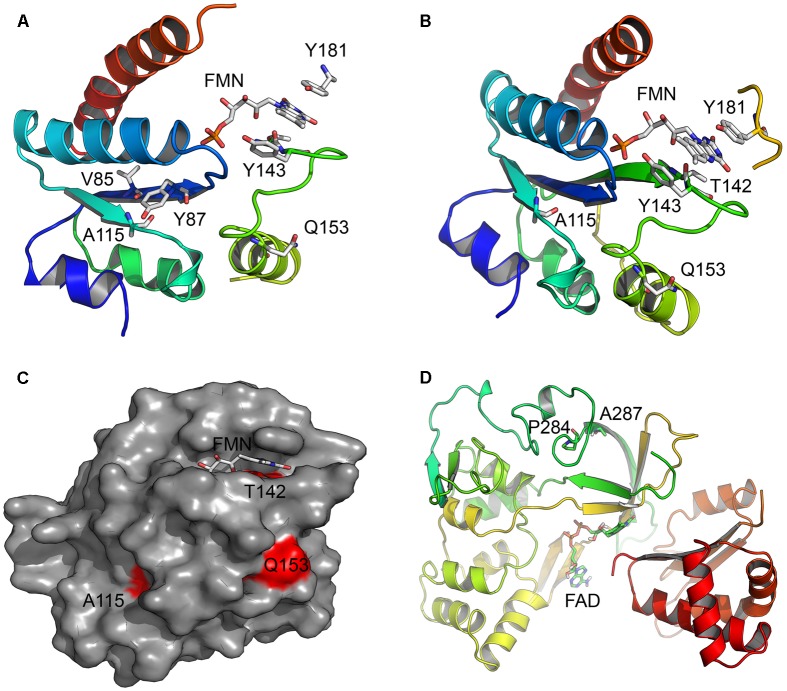
Location of amino acid changes in POR described in current study. The x-ray crystal structures of the human POR (NCBI# NP_000932) protein; the FMN binding domain (**PDB#** 1B1C) and the flexible hinge region (**PDB**# 3FJO) showing the location of mutations studied in this report. **(A)** Location of residue A115. Structure analysis revealed hydrogen bonding interaction between A115 and V85 and Y87 residues located on a neighboring beta sheet in the FMN binding domain of POR, implicating its role in structural stability. The models are colored in rainbow colors with violet at the N-terminus and red at the C-terminus. The co-factors FMN and FAD are shown as sticks. **(B)** Location of residues T142 and Q153. The mutation T142A is located next to Y143 residue crucial for FMN binding to POR and its mutation affected FMN binding. The Q153 residue is located at the surface of POR and predicted to have a role in the interactions with the redox partners. **(C)** A surface representation of the POR FMN binding domain showing mutated residues in red. The Q153 residue is exposed to surface, while residues A115 and T142 are also partially exposed. **(D)** Location of P284 residue in the hinge region of the POR. Hinge region is required for conformational flexibility and mutations in this region are predicted to cause conformational changes that would alter both the electron transport and the interaction with redox partners.

Aromatase catalyzes the conversion of androstenedione to estrone (E1), testosterone to estradiol (E2) and 16-hydroxytestosterone to estriol (E3) (Simpson et al., 1994) (**Figure 1**). The CYP19A1 reaction requires multiple interactions with POR, and therefore, changes in redox partner binding sites on either CYP19A1 or POR may alter enzymatic activities. To test this hypothesis, we selected several mutations located in the FMN binding domain and the hinge region of POR, which facilitate interactions with partner proteins. One of the variants studied here, the A115V was first identified in a patient (Huang et al., 2005). The patient harboring A115V mutation was from Caucasian background and had Beare–Stevenson syndrome, which results in skeletal as well as genital abnormalities (Przylepa et al., 1996; Huang et al., 2005). A rugated labia majora and anteriorly placed anus had been reported but no steroid or biochemical analysis were available (Huang et al., 2005). Structure analysis of A115V mutation revealed hydrogen bonding interaction between A115 and V85 and Y87 residues located on the neighboring beta sheet, implicating its role in structural stability (**Figure 7A**). Previously Agrawal et al. (2008) have shown that A115V mutation caused complete loss of CYP1A2 and CYP2C19 activities, while only a 20–30% loss of 17-hydroxylase and 17,20 lyase activities was observed in CYP17A1 assays (Huang et al., 2005). Our results indicate the severe loss of CYP19A1 aromatization activity could lead to abnormal steroid metabolism in patients with A115V mutation in POR gene. A detailed steroid analysis of such patients would be necessary to confirm this linkage.

The patient with T142A mutation was from Iraqi/Yemeni parents and did not have bone malformation but showed abnormal genitalia (Augarten et al., 1992). The ACTH stimulation test for the adrenal function indicated combined CYP17A1 and CYP21A2 deficiencies, but no genetic defects in these genes were found. Augarten et al. (1992) had hypothesized that a deficiency in another enzyme, likely a flavoprotein may be the cause but this was not pursued further, and only in 2005 the T142A mutation in the POR from this patient was identified (Huang et al., 2005). In previous reports a 97% loss of CYP1A2 activity and complete loss of CYP2C19 activity has been reported by Agrawal et al. (2008) for the T142A mutation, while a 40–50% loss of CYP17A1 activity was observed by Huang et al. (2005). The patient with T142A mutation of POR had low serum levels of testosterone; and androstenedione and dehydroepiandrosterone sulfate were low (but present) and did not improve upon ACTH stimulation, consistent with loss of CYP17A1 activities (Augarten et al., 1992). This is consistent with crucial physiological role of androgen regulating enzyme CYP17A1, where complete loss of activities is lethal. The mutation T142A (*POR^∗^12*) is located next to the Y143 residue which is crucial for the FMN binding to POR, and its mutation may affect FMN binding (**Figure 7B**). Consistent with this we found a severe loss of flavins in the T142A variant of POR (**Figure 4**). Loss of flavins could also make subtle conformation changes in POR and as these changes would be in the FMN binding domain of POR which interacts with redox partners via shape as well as charge-based protein–protein interactions, a change in activities of redox partners is expected. Severe loss of both CYP19A1 as well as CYP3A4 activities due to T142A mutation indicates a major impact on both steroid and drug metabolism in the patients carrying this mutation.

The mutation Q153R was first described from an Algerian family and was the product of an abortion carried out at 22 weeks of gestation due to information from an ultrasonographic examination which showed craniosynostosis with bilateral radiohumeral synostosis, bowing of long bones, and arachnodactyly (Huang et al., 2005). The karyotype of the fetus was 46,XY, and no abnormalities in external genitalia were detected. Steroids were not measured, but, since ABS was suspected, a GC-MS analysis of liver tissue was performed which indicated “substantially increased” lanosterol and dihydrolanosterol. In previous studies, the Q153R (*POR^∗^13*) variant of POR was found to retain only 25–30% of WT activity in assays with CYP17A1 (Huang et al., 2005) but has shown higher activities in other assays (Agrawal et al., 2008). A higher level of activity with CYP19A1 observed with this mutation would not indicate any damaging effect on a 46,XY fetus, but GC-MS analysis indicates this mutation may affect CYP51A1 activity. The Q153 residue is located at the surface of POR and may have a direct role in the interactions with the redox partners (**Figure 7C**). However, higher levels of activities observed in both the CYP19A1, as well as the CYP3A4 assays indicate that the impact of Q153R variant may be selective for individual P450 enzymes with some being adversely affected while others would be activated due to potentially better protein–protein interactions. The activating effect of Q153R variant of POR can be useful for potential biotechnological applications where commercial production of expensive chemicals through P450 mediated biotransformation is the preferred method of manufacturing.

The last mutation studied in this report, the P284L (rs 72557938), is located in the hinge region of POR, that is important for domain movements and efficient interactions between FMN and FAD binding regions and for the transfer of electrons from FAD to FMN (**Figure 7D**). In the sequencing study by Huang et al. (2008) the P284L mutation was present at an allele frequency of 0.003 in Chinese American population and in the currently available ExAc dataset containing 121000 samples it is present at an allele frequency of 0.00004132. A 54% loss of 17-hydroxylase activity was reported by Huang et al but the 17,20 lyase activity of CYP17A1 was 82% of the WT POR (Huang et al., 2008). No loss of flavin content was observed in the P284L variant which indicates altered inter as well as intra molecular protein–protein interactions may be responsible for the loss of activities in both the CYP19A1 as well as the CYP3A4 assays. Since the hinge region of POR is important for the domain motions to bring the FMN closer to the FAD for the transfer of electrons, a change in this region may affect the efficiency of electron transport process in POR, and therefore, affect the activities of partner proteins. It is possible that some redox partners of POR may experience greater impact on their catalytic activities while others may retain close to WT activities depending on their mode of interaction with POR.

In these studies, we have used POR and P450 proteins embedded in liposomes and removed the detergents used for solubilization of POR and P450s by treatment with biobeads (Bio-Rad Corp., Hercules, CA, United States) which provided a robust assay system. These results also emphasize the need for assay of POR variants with different partner proteins and substrates to accurately determine the impact of individual variations. The major polymorphism observed in POR is A503V (rs 1057868, *POR^∗^28*), which is present in about 27% of all alleles and shows wide variability across different populations (Caucasian and Hispanic populations: 31%; Pacific Islanders: 48%; Asian populations 35%; Japanese, 40%; African Americans 16%) (Burkhard et al., 2017). There is some evidence of *POR^∗^28* allele influencing the activities of drug metabolizing enzymes but more direct evidence is needed before any firm conclusions can be drawn (Pandey and Flück, 2013; Pandey and Sproll, 2014). As seen with the P284L mutation, there may be variations of POR in apparently normal populations with potentially disease causing effects. Since POR variations have now been linked to the metabolism of several drugs and steroids, it may be prudent to include the sequencing of POR gene while looking for genetic causes of altered drug or steroid metabolism. A detailed examination of the polymorphic variants of POR would be required to estimate their damaging effects.

## Author Contributions

Participated in research design: SU, SP, and AP. Conducted experiments: SU and SP. Contributed new reagents or analytical tools: NK. Performed data analysis: SU, SP, and AP. Overall supervision of the project: AP. Wrote or contributed the writing of the manuscript: SU, SP, NK, and AP.

## Conflict of Interest Statement

The authors declare that the research was conducted in the absence of any commercial or financial relationships that could be construed as a potential conflict of interest.

## Footnotes

1 www.rcsb.org

2 www.pymol.org

3 www.povray.org
